# Accuracy of script concordance tests in fourth-year medical students

**DOI:** 10.5116/ijme.5898.2f91

**Published:** 2017-02-25

**Authors:** Saad Nseir, Ahmed Elkalioubie, Philippe Deruelle, Dominique Lacroix, Didier Gosset

**Affiliations:** 1 University of Lille, School of Medicine, Lille, France; 2 University Hospital of Lille, Critical Care Center, Lille, France

**Keywords:** Script concordance tests, evaluation, fourth-year medical year, France

## Abstract

**Objectives:**

This investigation aimed to determine the validity of script concordance test (SCT), compared with clinical-case-related short-answer management problems (SAMP), in fourth-year medical students.

**Methods:**

This retrospective study was conducted at the Medical School of Lille University. Cardiology and gynecology examinations both included 3 SCT and 2 clinical-case-related SAMP.  Final score did not include SCT results, and was out of 20 points. The passing score was ≥10/20. Wilcoxon and McNemar tests were used to compare quantitative and qualitative variables, respectively. Correlation between scores was also analyzed.

**Results:**

A total of 519 and 521 students completed SAMP and SCT in cardiology and gynecology, respectively. Cardiology score was significantly higher in SCT than SAMP (mean ± SD 13.5±2.4 versus 11.4±2.6, Wilcoxon test, p<0.001). In gynecology, SCT score was significantly lower than SAMP score (10.8±2.6 versus 11.4±2.7, Wilcoxon test, p=0.001). SCT and SAMP scores were significantly correlated (p <0.05, Pearson’s correlation). However, percentage of students with SCT score ≥ 10/20 was similar among those who passed or failed cardiology (327 of 359 (91%) vs 146 of 160 (91%), χ^2^=0.004, df =1, p=0.952), or gynecology (274 of 379 (65%) vs 84 of 142 (59%), χ^2^=1.614, df=1, p=0.204) SAMP test. Cronbach alpha coefficient was 0.31 and 0.92 for all SCT and SAMP, respectively.

**Conclusions:**

Although significantly correlated, the scores obtained in SCT and SAMP were significantly different in fourth-year medical students. These findings suggest that SCT should not be used for summative purposes in fourth-year medical students.

## Introduction

Script concordance tests (SCT) assess clinical reasoning expertise in a context of uncertainty.^1^ Such uncertainty could result from missing information at the time of decision-making, or absence of evidence-based medical recommendations.

In spite of some format similarities, SCT differ from content-enriched, multiple choice questions (MCQ). Although MCQ deal with clinical reasoning end-point, or relevant knowledge, SCT assess some parts of the cognitive process. In MCQ, one has to choose a single best answer, whereas in SCT students are evaluated by agreement or concordance of their answers with those of an expert panel. Furthermore, MCQ add unnecessary complexity to factual knowledge, while SCT are a genuine simulation of patients’ clinical history without additional complexity.^2^

Recently, universities worldwide have used SCT for clinical reasoning in various medical disciplines including pediatric medicine,^3^ emergency medicine,^4^ critical care,^5^ anesthesiology,^6^ surgery,^7^ radiology,^8^ and other medical specialties.^6,9–11^ SCT is generally used for training and evaluation during the postgraduate medical studies. Previous studies have suggested that SCT could be used as a standardized instrument to evaluate growth in clinical reasoning skills.^12^^,^^13^ However, one of the limitations of using SCT in this context is the difficulty to give a clear and helpful feedback. Further, evidence supporting the validity of SCT scores with respect to examinees’ thought and response processes is still limited,^14^ and potential weaknesses of SCT have recently been outlined.^15^ See et al.^16^ analyzed SCT, and MCQ scores on pulmonary and critical care medicine tests in 16 fellows and 10 residents. They concluded that SCT was vulnerable to the intentional avoidance of extreme responses. Another recent study evaluated the judgment of a panel of emergency medicine consultants against evidence-based likelihood ratios regarding the diagnosis value of selected clinical and para-clinical findings in the context of an SCT.^4^ The results raised concerns regarding whether the judgments of an expert panel are sufficiently valid as the reference standard for this test. Moreover, SCT could be very difficult to construct, apply and correct. Roberti et al.^17^ suggested that these difficulties might make application of a SCT assessment method unfeasible in units with limited resources.

Several studies have assessed SCT feasibility and efficacy as an evaluation tool in fourth-year medical students.^18–26^ However, only few have compared SCT to other examination forms in the same group of students.^18,20–22^ Furthermore, these studies included few students. Given the above-discussed limitations of using SCT to assess medical students in routine, we hypothesized that SCT would not be accurate for summative purposes in fourth-year medical student, independently of the domain of knowledge. Therefore, we conducted this study to evaluate SCT validity, compared with SAMP, in assessment of fourth-year medical students.

## Methods

### Study design and participants

This retrospective study was conducted, in January 2013, at the Medical School of Lille University.The study was approved by the local Institutional Review Board (Comité de Protection des Personnes Nord-ouest IV).  Because of the retrospective observational design of the study, and in accordance with the French law, written informed consent was not required by the local IRB. All data were analyzed anonymously. Five hundred and twenty one students attending the fourth year of medical school were included in this study.

### Data-collection method and procedure

Students had received a dedicated training for SCT, including 2 hours of theory about definition and construction of SCT, and several practices during cardiology and gynecology practical teaching.  SCTs were constructed according to the guidelines of Dory et al.^2^ For each of cardiology and gynecology, two faculty members wrote the SCT. Both cardiology and gynecology SCT were reviewed and answered by 12 and 10 experts, respectively. Each SCT (3 in cardiology, and 3 in gynecology) included a clinical vignette and 3 hypotheses (or items).  Additional information was provided after each hypothesis. The questions pertained to the effect of the new piece of information on the initial hypothesis. Students provided their answers on a 5-point Likert scale (-2 to +2) (Appendices 1 and 2). SCT was rated for out of 20 (2.25 for the first 8 items, and 2 for the last item).

Cardiology and gynecology full tests lasted 2h30 each, and included 3 SCT and 2 clinical-case-related SAMP that were given to students at the beginning of the test. The cardiology and gynecology SAMP included two clinical cases with 8-10 questions, requiring open and short answers. These questions dealt with a clinical issue or the recall of factual knowledge. SAMP have been used in our Medical School for summative assessment for several years. An example of SAMP is presented in Appendix 3. The final score was out of 20 points for both cardiology and gynecology, and was calculated as the total of SAMP grades. The passing score was ≥10/20. SCT results were not included in the final score.

### Statistical analysis

SPSS software (IBM Statistics 22) was used for statistical analysis. Qualitative variables are presented as number (%). Distribution of quantitative variables was tested using Kolmogorov-Smirnov test. These data are presented as mean ± SD, as they were normally distributed.  Statistical significance was set at p-value < 0.05. Cronbach’s α coefficient computing was used to assess reliability of SCT and SAMP.

Scores of SCT and SAMP were compared, for cardiology and gynecology, using Wilcoxon test. The percentage of students with an SCT score ≥ 50% in the 2 groups of students who passed and failed the test was compared using McNemar test. Wilcoxon and McNemar tests are usually used to compare quantitative and qualitative data in the same individuals, respectively. Correlation between SCT score and final score was analyzed with the Pearson’s coefficient. 

## Results

Among the 521 included students, 265 (50.9%) were female. Their mean (± SD) age was 23.9 (±1.5) years, and the mean study year (± SD) was 4.6 (±1.1). Cronbach α coefficient was 0.31, and 0.92 for all SCT, and SAMP; respectively. Although 519 students completed SCT in cardiology, all students completed SCT in gynecology.

### Cardiology examination

A total of 519 students completed the 2 SAMP and the SCT in cardiology. Mean score was significantly higher in SCT compared with SAMP (13.5±2.4 vs 11.4 ± 2.6, Wilcoxon test, p <0.001). A score ≥ 50% of maximum score, i.e. ≥ 10/20, was significantly more frequent in SCT than in SAMP (473 students [91%] vs 359 students [69%], respectively, McNemar test, p< 0.001).

Percentage of students with a SCT score ≥ 10/20 was similar (χ^2^=0.004, df = 1, p=0.952) in the 2 groups of students who passed (final score ≥ 10/20, 327/359 [91%]) or failed (final score < 10/20, 146/160 [91%]) SAMP test.  SCT score was significantly correlated with SAMP score (Pearson’s correlation, r^2^=0.57, p=0.047).

### Gynecology examination

A total of 521 students completed the 2 SAMP and the SCT in gynecology. Mean score was significantly lower in SCT compared to SAMP (10.8 ± 2.6 vs 11.4 ± 2.7, Wilcoxon test, p=0.001).

A score ≥ 50% of maximum score, i.e. ≥ 10/20, was found significantly less in SCT than in SAMP (331 [63%] vs 379 [72%], McNemar test, p=0.001). Percentage of students with an SCT score ≥ 10/20 was similar (χ^2^=1.614, df =1, p = 0.204) in the 2 groups of students who passed (final score ≥ 10/20, 247/379 [65%]) and failed (final score ≤ 10/20; 84/142 [59%]) SAMP test.

SCT score was significantly correlated with SAMP score (Pearson’s correlation, r^2^=0.92, p=0.004). 

## Discussion

Our results show a significant correlation between SCT and SAMP scores. However, these scores were significantly different. Furthermore, percentage of students with an SCT score ≥ 10/20 was similar in the 2 groups of students who passed and failed the examination, based on the SAMP score. These results suggest that SCT failed in differentiating strong from weak students based on SAMP scores.

To our knowledge, our study is the first to compare SCT and SAMP in a large cohort of fourth-year medical students. In a cohort of 85 fourth-year medical students, Jouneau et al. evaluated SCT as a tool for assessment of clinical reasoning and knowledge organization in pulmonology clinical cases written examination.^18^ Students’ score in clinical cases and SCT were significantly correlated, as in our study. However, these 2 studies differ in several aspects. Our study included a larger number of students, as compared with the study of Jouneau et al. (519 vs. 85). It also dealt with two medical disciplines (cardiology and gynecology) rather than one (pulmonology), thus allowing more relevant generalization of its results. Furthermore, whilst SCT were compared to SAMP in our study, Jouneau et al. compared SCT with clinical cases.

Another recent study evaluated the utilization of SCT as an assessment tool for fifth-year medical student in rheumatology. The test included 60 questions, and was administered to a panel of 19 experts, and to 26 students.^27^ Fifteen students completed SCT in its entity, and had a mean score of 61.5.  Despite the low participation rate, the possibility of using this internet-based SCT was demonstrated.

Several studies compared the performance of SCT and MCQ in students’ assessment. Fournier et al. compared SCT and content-enriched MCQ performance in assessment of clinical reasoning expertise in the field of emergency medicine.^28^ In spite of adequate Cronbach α coefficient (ranging from 0.85-0.95), SCT and MCQ were not significantly correlated (r^2^ = 0.016, p = 0.59). As further pointed out by the authors to explain these negative results, only few students, and physicians were included in this study (20 first-year residents, 16 sixth-year medical students, and 7 certified doctors). Collard et al.^22^ compared SCT with factual knowledge test scores (true/false test with a 0-100% ascertainment degree), by 104 3rd, 4th, 5th, and 6th year medical students, and found a significant correlation between the 2 tests. Brailovsky et al.^21^ also found SCT to be significantly correlated to SAMP in a cohort of 24 medical students, in Quebec, from the end of their clerkship to the end of their residency.

In a recent study, Kelly et al.^20^ compared reliability, validity and learner satisfaction between SCT, MCQ and National Board of Medical Examiners tests. This study included 120 3rd and 4th year medical students who were given 20-item SCT and MCQ. SCT examination was more valid than the MCQ examination because of better correlation with clinical performance. However, SCT was initially less reliable and less preferred by students.

Despite the significant correlation found in our study between SCT and SAMP scores, the scores obtained in these tests were significantly different. This is most likely due to different type of knowledge assessed by SCT and SAMP. In fact, SCT assess clinical reasoning expertise in a context of uncertainty, whereas SAMP assesses clinical situation-based factual knowledge. One could argue that whilst SAMP is valuable for summative assessment of students, SCT would allow better ranking of students. However, our results suggest that SCT should not be used for summative assessment. Van den Broek et al.^29^ reported similar conclusions in final-year medical students.

One of the strengths of our study is the fact that SCT were not valid in summative assessment in two different specialties, i.e. cardiology and gynecology. No clear difference was found in the format of SCT in cardiology and gynecology to explain the better scores obtained in cardiology compared with gynecology. One potential explanation for this discrepancy is the clinical experience of students.

Our study has several limitations. The direct comparison of similar concepts between SCT and SAMP was not possible, as detailed learning objectives were not available. In addition, students knew that the SCT would not be taken into account in their final grade, and this might have reduced their efforts in that section of the test. However, the students knew that SCT would probably be used for their final examination at the final year of medical studies. Another limitation of our study lies in its reliability, with an SCT Cronbach α coefficient of only 0.31. Some authors have reported an adequate reliability with a minimum of 15 experts. Accordingly, the 12 and 10-member expert panels could be considered relatively small, and might have negatively affected Cronbach α.^30^^,^^31^ Furthermore, few SCT hypotheses (n=3) did not allow consensus among experts who answered the SCT. Nevertheless, exclusion of these conflicting questions from statistical analysis did not improve Cronbach α coefficient (results not shown). Absence of consensus among experts is one the major limitations of SCT, as no clear action is recommended when experts disagree.  Some authors suggest that Cronbach’s alpha might not be the best way to assess SCT reliability, as clinical reasoning may not be a unitary concept. Finally, our results could not be generalized because of the single center design, the fact that SCT were only evaluated in cardiology and gynecology, and the low Cronbach’s α. Further multicenter studies are required to confirm our findings.

## Conclusions

Although significantly correlated, SCT and SAMP scores of cardiology and gynecology were significantly different in fourth-year medical students. SCT failed in differentiating strong from weak students, based on SAMP scores. These results suggest that SCT should not be used for summative purposes in fourth-year medical student.

### Acknowledgements

We thank Dr Laura Ravasi for her assistance in writing and English editing the manuscript, on behalf of the University of Lille, France. 

### Conflict of Interest

The authors declare that they have no conflict of interest.

## Supplementary material

Supplementary file 1 Appendix 1. An example of a cardiology SCT case vignette

Supplementary file 2 Appendix 2. An example of a Gynecology SCT case vignette

Supplementary file 3 Appendix 3. Example of Gynecology short answer management problem
