# Supplementary material for: Accuracy of script concordance tests in fourth-year medical students
Source: Int J Med Educ. 2017 Feb 25;8:63–9. doi: 10.5116/ijme.5898.2f91 (PMC5339020; doi:10.5116/ijme.5898.2f91)
Supplement: Supplementary file 1 — Appendix 1. An example of a cardiology SCT case vignette [file ijme-8-63-S1.pdf]

Appendix 1

An example of a cardiology SCT case vignette

A 50-year old man with a history of myocardial infarction with normal ejection fraction is admitted to emergency because of syncopal attacks.

You are thinking of the following hypothesis:	And then the ECG shows	You would then consider the hypothesis to be:
An intermittent atrioventricular conduction block	A left bundle branch block	-2 -1 0 + 1 2
An orthostatic hypotension	A first-degree atrioventricular conduction block	-2 -1 0 + 1 2
A sinus dysfunction	An atrial fibrillation	-2 -1 0 + 1 2

Please make your choice of the most adequate answer (on the answer data sheet): The hypothesis would become

-2	much less likely (= ruled out or almost ruled out)
-1	less likely
0	neither more nor less likely
+1	more likely
+2	much more likely (= certain or almost certain)
