# Supplementary material for: Accuracy of script concordance tests in fourth-year medical students
Source: Int J Med Educ. 2017 Feb 25;8:63–9. doi: 10.5116/ijme.5898.2f91 (PMC5339020; doi:10.5116/ijme.5898.2f91)
Supplement: Supplementary file 2 — Appendix 2. An example of a Gynecology SCT case vignette [file ijme-8-63-S2.pdf]

Appendix 2

An example of a Gynecology SCT case vignette

A nulliparous 22-year old woman is admitted to emergency because of pelvic pain and moderate metrorrhagia, after 5 weeks of amenorrhea. Her menstrual cycles are regular and she is not taking any contraception, as she is heavy smoker (20 cigarettes per day).

You are thinking of the following hypothesis:	And then you find that	You would then consider the hypothesis to be:
An extra uterine pregnancy	Abdominal and pelvic echography is normal	-2 -1 0 + 1 2
An extra uterine pregnancy	Chlamydia serology is positive	-2 -1 0 + 1 2
A spontaneous miscarriage	The uterus is empty on echography	-2 -1 0 + 1 2

Please make your choice of the most adequate answer (on the answer data sheet): The hypothesis would become:

- -2 much less likely (= ruled out or almost ruled out)
- -1 less likely
- 0 neither more nor less likely
- +1 more likely
- +2 much more likely (= certain or almost certain)
