# Supplementary material for: Accuracy of script concordance tests in fourth-year medical students
Source: Int J Med Educ. 2017 Feb 25;8:63–9. doi: 10.5116/ijme.5898.2f91 (PMC5339020; doi:10.5116/ijme.5898.2f91)
Supplement: Supplementary file 3 — Appendix 3. Example of Gynecology short answer management problem [file ijme-8-63-S3.pdf]

Appendix 3

Example of Gynecology short answer management problem

A 19-year old patient is consulting for a 4-month long amenorrhea. She has no medical history, and has never been on oral contraception.

Question	Response	Note
1. What is your first diagnosis?	Pregnancy	1
2. Which elements should be searched for during medical interrogatory and clinical examination?	Nausea, mammary tension, mastodynea, pelvic heaviness<br>Red uterine cervix, Le Noble's sign	1
3. The first diagnosis was eliminated by interrogatory and laboratory examination. The patient describes a voluntary weight loss of 10 kg, with a BMI of 16 kg/m^2. What is your diagnosis?	Functional hypothalamic amenorrhea (or mental anorexia)	1
4. Which test should be performed to confirm this hypothesis? What is the objective of this test?	-Progesterone test<br>-Differentiate hypoestrogenic amenorrhea from normoestrogenic amenorrhea	1
5. Which hormonal analyses should be performed? Which results are expected in this case?	FSH (normal or low)<br>LH (low)<br>Estradiol (low or very low)<br>Prolactin (normal)	1
6. Which morphologic examination should be performed? And why?	Hypothalamic and hypophyseal MRI<br>To eliminate a tumoral process	1
7. You see the patient six years later, she gained 25kg, and her BMI is 23 kg/m^2. She has now irregular menstrual cycles, excessive hair (rated 10 on Friedmann and Gallwey scale), and suffers acne. What is your diagnosis?	Polycystic ovarian syndrome	1
8. What is the pathophysiologic primitive abnormality responsible for this disease?	Abnormality in thecal cells resulting in increased androgen production	1
9. Which hormonal analyses should be performed? Which results are expected in this case?	FSH (normal)<br>LH (normal or increased)<br>Estradiol (normal)<br>Androgen (normal or slightly increased)<br>Procalcin (normal)<br>Free urinary cortisol (normal)	1
10. Which morphologic examination should be performed? Under which conditions? And what are the expected results?	Pelvic ultrasonography<br>At the beginning of the folicular phase<br>Polycystic ovaries, at least 12 follicles per ovary, increased volume and surface of ovaries, normal endometer	1

Abbreviations: BMI, body mass index; FSH, Follicle stimulating hormone; LH, luteinizing hormone; MRI, magnetic resonance imaging
